# Health Promotion Among Mexican-Origin Survivors of Breast Cancer and Caregivers Living in the United States–Mexico Border Region: Qualitative Analysis From the Vida Plena Study

**DOI:** 10.2196/33083

**Published:** 2022-02-24

**Authors:** Meghan B Skiba, Melissa Lopez-Pentecost, Samantha J Werts, Maia Ingram, Rosi M Vogel, Tatiana Enriquez, Lizzie Garcia, Cynthia A Thomson

**Affiliations:** 1 Biobehavioral Health Science Division College of Nursing University of Arizona Tucson, AZ United States; 2 Department of Clinical Translational Sciences College of Medicine University of Arizona Tucson, AZ United States; 3 Department of Health Promotion Sciences Mel and Enid Zuckerman College of Public Health University of Arizona Tucson, AZ United States; 4 Mariposa Community Health Center Nogales, AZ United States

**Keywords:** Mexican-origin Hispanics, breast cancer, survivorship, caregivers, border health, lifestyle, diet, physical activity, health promotion, mobile phone

## Abstract

**Background:**

Hispanic survivors of cancer experience increased cancer burden. Lifestyle behaviors, including diet and physical activity, may reduce the cancer burden. There is limited knowledge about the posttreatment lifestyle experiences of Hispanic survivors of cancer living on the United States–Mexico border.

**Objective:**

This study aims to support the development of a stakeholder-informed, culturally relevant, evidence-based lifestyle intervention for Mexican-origin Hispanic survivors of cancer living in a border community to improve their dietary quality and physical activity.

**Methods:**

Semistructured interviews with 12 Mexican-origin Hispanic survivors of breast cancer and 7 caregivers were conducted through internet-based teleconferencing. The interviews explored the impact of cancer on lifestyle and treatment-related symptoms, perception of lifestyle as an influence on health after cancer, and intervention content and delivery preferences. Interviews were analyzed using a deductive thematic approach grounded in the Quality of Cancer Survivorship Care Framework.

**Results:**

Key survivor themes included perception of Mexican diet as unhealthy, need for reliable diet-related information, perceived benefits of physical activity after cancer treatment, family support for healthy lifestyles (physical and emotional), presence of cancer-related symptoms interfering with lifestyle, and financial barriers to living a healthy lifestyle. Among caregivers, key themes included effects of the cancer caregiving experience on caregivers’ lifestyle and cancer-preventive behaviors and gratification in providing support to the survivors.

**Conclusions:**

The interviews revealed key considerations to the adaptation, development, and implementation of a theory-informed, evidence-based, culturally relevant lifestyle program to support lifestyle behavior change among Mexican-origin Hispanic survivors of cancer living in border communities. Our qualitative findings highlight specific strategies that can be implemented in health promotion programming aimed at encouraging cancer protective behaviors to reduce the burden of cancer and comorbidities in Mexican-origin survivors of cancer living in border communities.

## Introduction

### Background

Cancer remains the leading cause of death among Hispanic people in the United States [1]. Cancer incidence rates are higher among Hispanic people compared with non-Hispanic White people for obesity-related cancers [1]. Obesity-related cancers have a metabolic etiology that can result in significant comorbidities. Many survivors of cancer experience comorbid conditions that may influence prognosis. Common comorbid conditions include cardiovascular disease, type 2 diabetes mellitus, hypertension, and obesity [2-4] and metabolic diseases that disproportionately affect Hispanic patients [5]. The burden of these comorbidities is higher among Hispanic people [6-8]. Among border-dwelling Hispanic people in the United States, mortality incidence rates exceed those in non-Hispanic White people for all obesity-related cancer types [9], which supports the need for health promotion programs to reduce health disparities in this vulnerable group.

Disparities in health are magnified in populations living along the United States–Mexico border. The border-dwelling population is largely of Mexican origin and is expected to double in size in the coming years [10]. Border communities are medically underserved and experience higher rates of poverty, poorer health outcomes, and lower access to professional health care than the general US population [10,11]. Breast cancer, an obesity-related cancer, is the most commonly diagnosed cancer type and the leading cause of cancer death among Hispanic women [1]. Although it is known that certain health behaviors are cancer protective, such as plant-dominant eating patterns and physical activity [12,13], previous research has shown that cultural health beliefs, social norms, access to resources, and cultural food preferences influence the health behaviors of Hispanic women [14,15]. Of note, compared with non-Hispanic White survivors of breast cancer, Hispanic survivors of breast cancer are more likely to report their health as fair or poor and are less likely to meet diet and physical activity recommendations [16,17].

Currently, there is limited knowledge of the cancer survivorship experience of Mexican-origin women, especially those living along the United States–Mexico border. The Quality of Cancer Survivorship Care Framework developed by Nekhlyudov et al [18] suggests that cancer survivorship quality is influenced by a variety of interrelated care needs, including management of physical and psychosocial symptoms related to treatment, control of comorbid conditions, and adoption of prevention-focused lifestyle behaviors. Health promotion interventions are poorly accessed by Hispanic populations [19,20], potentially because the components of these interventions lack relevance [21,22]. Culturally adapted, stakeholder-informed interventions are more successful in reaching high-risk, underserved populations [23,24]. Barriers to intervention adoption include language accessibility, lack of nutrition education constructed around culturally-based foods, and poor access to resources [22]. As compared with non-Hispanic White women, Mexican-origin Hispanic survivors of breast cancer have been shown to have poorer health-related quality of life and poorer adherence to diet and physical activity recommendations [16,17]; it is important to determine aspects to include in lifestyle interventions that can more appropriately meet the unique needs of Mexican-origin Hispanic survivors of cancer. Stakeholder input is central to this process.

### Objectives

Developing and adapting a lifestyle intervention that includes components that are culturally relevant can increase the acceptability of health promotion programs for women living along the United States–Mexico border and more effectively help promote cancer protective behaviors, specifically dietary quality and physical activity, ultimately improving health outcomes. The aims of this qualitative study are to (1) determine the important aspects for developing and adapting a culturally relevant lifestyle intervention for survivors of cancer in border communities and 2) contribute to a greater understanding of lifestyle behavior and survivorship in Mexican-origin breast survivors of cancer and their caregivers.

## Methods

### Overview

This qualitative study is part of Vida Plena, a community-based participatory research study [25,26] between the Mariposa Community Health Center (MCHC) and the University of Arizona Cancer Prevention and Control Research Network. MCHC is a designated federally qualified health center in Nogales, Arizona, United States, which provides primary care services to nearly half of all residents of Santa Cruz County, Arizona, United States. Nogales, Arizona, United States, is contiguous with the international border with Mexico. Its way of life—history, people, culture, and economy—is linked to its neighbors in Sonora, Mexico. Nogales, the largest city in Santa Cruz County, is largely of Hispanic (94.5%) and Mexican origin [27]. Community health workers (CHWs), who are frontline workers and have a close relationship with the community [28], are central to the MCHC health promotion efforts, including cervical and breast cancer screening, navigation services for cancer treatment, and supportive care after treatment. Breast cancer is the most common cancer type among women served by MCHC. MCHC CHWs have facilitated a breast cancer support group for >20 years in the Nogales community. The cancer support group participants developed a strong network of mutual support and provided ongoing guidance and input for the project.

### Participants

The MCHC CHWs (TE and LG) currently facilitating the cancer support network engaged cancer support group members in creating the program name *Vida Plena*, derived from *vivir una vida plena*, meaning to live a full life. This name resonated with survivors of cancer because they maintained that life was much more than their cancer diagnosis. Vida Plena was used to identify all the study-related materials. To gain additional perspectives on survivorship experience, this study included both survivors of cancer and their caregivers.

For survivors of cancer, participants were eligible to participate in the study if they were of Hispanic origin, aged ≥18 years, had a history of breast cancer, were female, and were diagnosed with cancer in the previous 15 years. For cancer caregivers, the eligibility criteria included self-identification as a caregiver for an individual with cancer. No sex restrictions were applied. MCHC CHWs recruited participants through referral by MCHC health care providers and CHWs, and electronic flyers delivered to a pre-existing cancer support group chat through *WhatsApp*. Eligible survivors of cancer were asked if their caregivers were interested in participating.

### Research Ethics Board and Informed Consent

MCHC CHWs (TE and LG) and research staff (RMV and MLP) recruited all participants and assisted with the informed consent process. Informed consent was obtained from all participants included in the study. All study materials were available in English and Spanish. Participants were reimbursed US $25 for their time. The study protocol was approved by the University of Arizona Institutional Review Board (2005660838). Study procedures were completed accordance with the ethical standards of the responsible committee on human experimentation and with the Helsinki Declaration of 1975, as revised in 2000.

### Data Collection

Owing to current public health recommendations at the time of this study related to reducing the spread of SARS-CoV-2 (COVID-19) and the exposure risk for survivors of cancer [29], all study activities, including recruitment, consent, interviews, analysis, and interpretation, were completed remotely via the internet and telephone. The study was conducted entirely in Spanish as the preferred language of participants. The existing relationship of the CHW with community members was essential to our success in overcoming barriers to recruitment to this internet-based interaction.

After enrollment, a bilingual and bicultural research staff member (RMV or MLP) contacted the participants via telephone to administer a short questionnaire on demographics and diet and physical activity behaviors before and after cancer treatment, adapted from the Women’s Healthy Eating and Living Study [30]. The research staff member scheduled a separate appointment for the semistructured interviews, which included 10 open-ended questions related to the impact of cancer on lifestyle, perceptions of lifestyle, and intervention content and delivery. The interviewer used probe cues to elicit deeper responses. The interviewer incorporated important cultural values that may influence lifestyle. Defined norms and roles in Mexican culture (eg, establishing warm interpersonal relationships, respect, and pleasant social exchanges) were applied during all the interviews [31]. The same interview guide was used to interview survivors and caregivers. The guide was developed with MCHC CHWs and is detailed in Table 1. All interviews were completed using Zoom software (Zoom Video Communication Inc) and were audio recorded, with permission, using integrated Zoom features for transcription and analysis. With Zoom, participants could complete the interview through the internet or telephone dial-in; video was disabled during internet-based calls to assure that all interviews relied on a consistent, audio response to queries. Each interview lasted approximately 1 hour.

**Table 1:** Interview question guide for Vida Plena.

Construct	Question (English)	Question (Spanish)	Probe
**Impact of cancer on lifestyle**			
	What does an average day look like for you?	¿Cómo es un día normal para usted?	Meals Social Family Work Routines Weekend vs weekday
	What helps you live a healthy lifestyle?	¿Qué le ayuda a vivir un estilo de vida saludable?	Facilitators
	How is your lifestyle different after cancer treatment?	¿Cómo es diferente el estilo de vida después del tratamiento del cáncer?	Nutrition or diet Physical activity or exercise
	What makes it difficult to live a healthy lifestyle?	¿Qué hace que sea difícil vivir un estilo de vida saludable?	Barriers Obstacles
	What things would make it easier for you to live a healthy lifestyle?	¿Qué le facilitaría an usted tener un estilo de vida saludable?	Support Resources
**Perceptions of lifestyle**			
	What, according to you, is a healthy lifestyle?	¿Qué significa para usted un estilo de vida saludable?	Importance of healthy eating and physical therapy after cancer treatment Change talk or thoughts around change
	How do you feel about diet or physical activity (lifestyle) as a way to reduce disease risk?	¿Qué piensa sobre la dieta/actividad física (estilo de vida) como una forma de reducir el riesgo de enfermedad?	Beliefs Attitudes Knowledge
**Intervention content and delivery**			
	How would you feel about receiving information and support for making healthy lifestyle choices?	¿Cómo se sentirá al recibir información y apoyo para elegir un estilo de vida saludable?	Preferences for information Type of support
	What is your preference for receiving information about healthy lifestyle behaviors?	¿Cuál es su preferencia para recibir información sobre comportamientos de estilo de vida saludable?	Written materials Web-based Telephonic Face to face Individual or group
	What are the reasons for you to choose (or not choose) to participate in a lifestyle program?	¿Cuáles son las razones por las cuales elegiría (o no elegiría) participar en un programa de estilo de vida?	Access Norms

### Data Analysis

Descriptive statistics were completed using STATA (version 16.1; StataCorp LLC). Audio recordings of interviews were transcribed using Google Speech-to-Text application programming interface (Alphabet Inc) with quality control by native Spanish speakers by the Behavioral Measurement and Interventions Shared Resource [32] at the University of Arizona Cancer Center. Authors (MLP and MI) coded the interviews using the Quality of Cancer Survivorship Care Framework [18] and developed codebooks for survivors and caregivers in consensus with MCHC partners. The thematic framework included five domains for cancer survivorship quality: health promotion (lifestyle and cancer-preventive behaviors), recurrences and new cancers (cancer screening practices), physical effects (symptoms resulting from cancer treatment), psychosocial effects (psychosocial symptoms, financial impact, and employment), and chronic conditions (non–cancer-related conditions). Verified transcripts were independently dual coded in Spanish by MLP and MI for relative themes and content using Dedoose 8.3.43 (SocioCultural Research Consultant LLC). After initial coding of the first 2 interviews, MLP and MI met to discuss definitions and resolve any discrepancies in coding. On the basis of this discussion, the code spirituality or religion was added to capture thematic references to the participants’ faith or spiritual beliefs. The remainder of the interviews were coded using the revised codebook. A directed content analysis approach was used to conceptualize a theoretical framework and highlight relationships [33].

**Figure 1:**
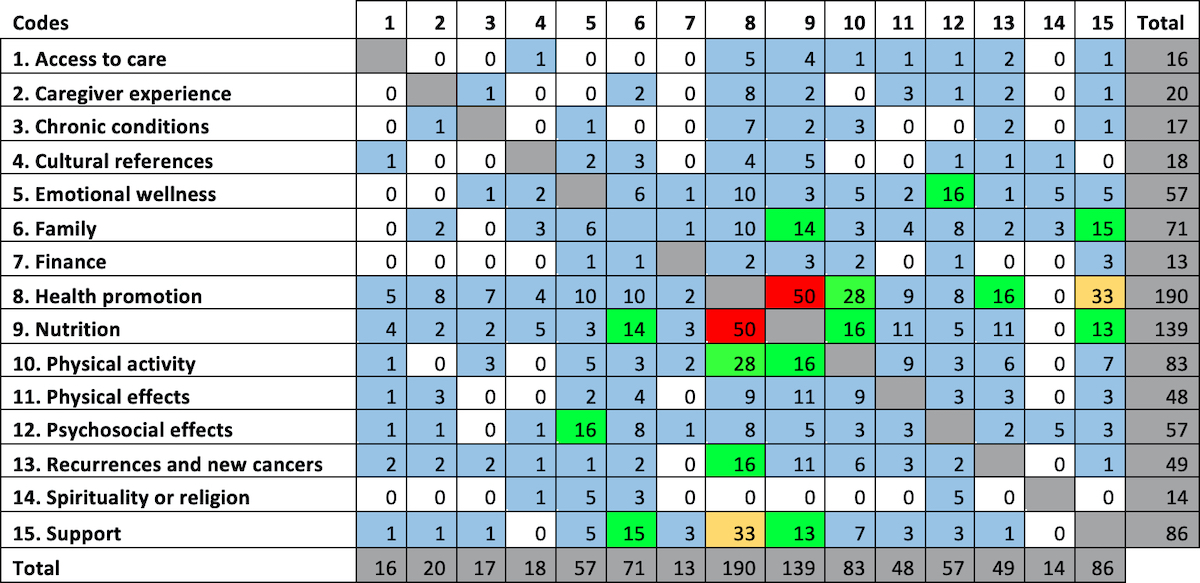
Theme overlap and co-occurrence among survivors of breast cancer and caregivers participating in Vida Plena. Numbers across the top row of the table correspond to the numbered theme listed in the first column. Numbers in each cell correspond to the count of code co-occurrences among themes. Color categories correspond to the count (white=0, blue=1-10, green=11-30, yellow=31-49, and red=50).

A participatory process for thematic analysis and interpretation with the MCHC included weekly meetings and discussions of themes throughout the analytic process with the entire research team. The coded domains interrelate and are often codependent on one another, and thus, subthemes that evolved in collaboration with MCHC CHWs included diet, physical activity, family, support, and finance. The 2 perspectives on survivorship (survivor and caregiver) were analyzed using the same framework. The overlap of themes is shown in Figure 1.

## Results

### Participants

A total of 26 individuals recruited by the CHWs were contacted for an interview (16/26, 62%, survivors; 10/26, 38%, caregivers). Of the 26 individuals, 3 (12%) were not eligible (2 survivors and 1 caregiver) and 2 (8%) caregivers dropped out after enrollment. Of the 21 participants, all of them (100%) completed the questionnaires (14 survivors and 7 caregivers) and 19 (91%) completed interviews (12 survivors and 7 caregivers).

All survivors of cancer were Spanish-speaking women, with a history of breast cancer. Most cancer caregivers were women and were daughters, mothers, or friends of the survivors. The average age of survivors of cancer was 57.4 (SD 12.4) years compared with 41.1 (SD 15.3) years for cancer caregivers. All participants were of Mexican origin, and most of the participants were Mexican-born. All survivors of cancer reported having at least one comorbid condition, with most of them having 4 or more. The most common comorbid conditions among survivors of cancer were vision problems (9/14, 64%), high cholesterol (6/14, 43%), and type 2 diabetes mellitus (4/14, 29%). The most common posttreatment symptoms included pain (6/14, 43%), sleep disorders (5/14, 36%), and depression or anxiety (4/14, 29%). Information on comorbid conditions experienced by cancer caregivers was not collected. Participant characteristics are detailed in Table 2. With regard to technology use, most survivors of cancer and caregivers used SMS text messaging and the internet several times a week or more, and all of them currently owned a smartphone (Table 3).

Many self-reported lifestyle behaviors of survivors of cancer and caregivers changed from before and after diagnosis (data not shown). After diagnosis, many survivors and caregivers reported increasing their intake of fruit (10/21, 48%), vegetables (10/21, 48%), fish (11/21, 52%), poultry (12/21, 58%), nuts (7/21, 33%), and whole grains (8/21, 38%) and decreasing their intake of red meat (14/21, 67%), fried food (14/21, 67%), fast food (8/21, 39%), and sweets (8/21, 39%) compared with before cancer diagnosis. The most commonly reported physical activities were walking (13/21, 62%) and dancing (13/21, 62%), with a low reported prevalence of mind-body physical activity (eg, Tai Chi and yoga; 2/21, 10%). Prayer was described as a form of meditation. For most participants, there was no change in physical activity after cancer diagnosis, with the exception of walking, which showed a 53% (11/21) increase.

**Table 2:** Demographics and health characteristics of participants enrolled in Vida Plena (N=21).

Demographics			Survivor (n=14)		Caregiver (n=7)		Total
Age (years), mean (SD)			57.4 (12.4)		41.1 (15.3)		52.0 (15.2)
Sex (female), n (%)			14 (100)		5 (71)		19 (90)
**Education, n (%)**							
	High school or less	7 (50)		4 (57)		11 (19)	
	Bachelor’s degree or higher	7 (50)		3 (43)		10 (38)	
Hispanic population^a^, n (%)			13 (100)		7 (100)		20 (100)
Mexican origin, n (%)			14 (100)		7 (100)		21 (100)
**Lived in current neighborhood (years), n (%)**							
	<10	5 (36)		4 (57)		9 (43)	
	≥10	9 (64)		3 (43)		12 (57)	
Spanish primary language spoken, n (%)			14 (100)		5 (71)		19 (90)
Currently married, n (%)			8 (57)		4 (57)		12 (57)
**Employment, n (%)**							
	None	12 (86)		3 (43)		15 (71)	
	Part time or full time	2 (14)		4 (37)		4 (19)	
**Cancer diagnosis, n (%)**							
	Breast	14 (100)		N/A^b^		N/A	
**Cancer stage at diagnosis, n (%)**							
	Stage 1	3 (21)		N/A		N/A	
	Stage 2	5 (36)		N/A		N/A	
	Stage 3	3 (21)		N/A		N/A	
	Not known	3 (21)		N/A		N/A	
Age diagnosed (years), mean (SD)			49.6 (12.4)		N/A		N/A
In active treatment, n (%)			7 (50)		N/A		N/A
Family history of cancer, n (%)			10 (71)		5 (71)		15 (71)
BMI (kg/m^2^), mean (SD)			28.8 (6.2)		29.7 (5.7)		29.1 (5.9)
Ever smoked, n (%)			3 (21)		3 (43)		6 (29)
Any comorbid condition, n (%)			14 (100)		N/A		N/A
**Count of comorbid conditions^c^, n (%)**							
	None	0 (0)		N/A		N/A	
	1-3	6 (43)		N/A		N/A	
	≥4	8 (57)		N/A		N/A	

^a^Missing data n=1.

^b^N/A: not applicable.

^c^Comorbidities include type 2 diabetes, asthma, depression or anxiety, heart disease, vision problems, arthritis, chronic gastrointestinal conditions, sleep disorder, high cholesterol, pain, and other comorbid conditions. Comorbidity data were not collected for cancer caregivers.

**Table 3:** Technology use among survivors of cancer and caregivers participating in Vida Plena (N=21).

Technology use		Survivor (n=14), n (%)	Caregiver (n=7), n (%)	Total, n (%)
Own a cell phone		14 (100)	7 (100)	21 (100)
Own a smartphone		13 (93)	7 (100)	20 (95)
**How often use SMS text messaging**				
	Rare to never	3 (21)	1 (14)	4 (19)
	Several times a week	2 (14)	2 (29)	4 (19)
	At least once a day	1 (7)	1 (14)	2 (10)
	Many times a day	8 (57)	3 (43)	11 (52)
**How often use the internet**				
	Rare to never	4 (29)	1 (14)	5 (24)
	A few times a month	1 (7)	0 (0)	1 (5)
	Several times a week	2 (14)	1 (14)	3 (14)
	At least once a day	1 (7)	1 (14)	2 (10)
	Many times a day	6 (43)	4 (57)	10 (48)

### Qualitative Results for Survivors

#### Health Promotion

##### Barriers Related to Healthy Eating

Many survivors of cancer reported that eating healthy was an important lifestyle habit for the prevention of cancer recurrence and the development of cancer in their family members. Survivors reported that among the barriers to healthy eating was the idea that traditional northern Mexican foods are unhealthy. Specifically, one survivor highlighted that Mexican dishes can be very greasy:

I believe that the biggest barrier in terms of the culture where I come from is the food that has been instilled in us, the style of food is very, very fatty sometimes, all Mexican dishes are very fatty

Survivor #1 (original quotes in Spanish are available in Multimedia Appendix 1)

In addition, participants alluded that eating healthy was difficult to do because of the lifestyle of the people around them. Specifically, food choices, time of meals, and food quantity were influenced by the participant’s home and social environment, “for me, one of the obstacles in my life is the lifestyle of the people with whom I live” (Survivor #16).

##### When and What Not to Eat

In total, 2 main themes emerged among survivors of cancer related to dietary changes and the adoption of healthier dietary habits. The first identified theme specific to dietary changes among participants was the concept of eating *on time*. Survivors consistently reported a belief that eating each meal at specific times of the day was an important aspect of healthy eating:

If you want to live a healthy life you should eat breakfast at exactly 8 in the morning, eat at 2 in the afternoon, and have dinner at 8 at night

Survivor #14

Second, there was a clear focus on specific foods to avoid or consume less. For example, survivors of cancer reported focusing efforts on staying away from or reducing the intake of canned foods, sugar, dairy, meat, and soy. Multiple participants reported reducing the number of tortillas and *harinas* (flour-based products) in their diet after diagnosis:

I think one should eat less of everything, for example for many years I have been eating just two tortillas and that is it

Survivor #2

Importantly, when speaking about dietary changes for a healthier lifestyle, survivors of cancer emphasized reducing or avoiding what they considered unhealthy foods but did not report the foods or food groups they felt they could eat more of to add to a healthy diet.

##### Access to Reliable Diet-Related Information

Survivors reported a lack of reliable diet information as a common barrier. Survivors of cancer narrated their personal experiences in accessing dietary information through the internet and the difficulty in identifying quality and reliable information. Survivors reported the need to have access to a nutrition professional during and after their cancer treatment

I have always thought that people who enter cancer treatment and survivors, just like they send us to physical therapy after our surgeries, they should also send us to a nutrition class or to the nutritionist

Survivor #1

Participants also expressed their desire for nutrition education not only for themselves, but for their families:

We have to re-educate the whole family...I have yet to find resources that provide support to the family to change the eating habits, therefore it is up to me to educate them

Survivor #1

##### Role of Physical Activity After Treatment

Survivors recognized the benefits of physical activity in overall health and for the prevention of cancer recurrence. Importantly, a common theme was the use of physical activity as a tool to manage physical effects after cancer treatment:

Exercise is now more necessary for me because my arm, well, you can see that I get numb, and if I am not exercising it becomes heavy and I feel swollen...then I have to exercise to remove that feeling

Survivor #11

The most commonly reported physical activity was walking with dancing and biking, similar to other reported activities.

##### Physical Activity Gradually Decreases Over Time

A common theme among survivors of cancer was recognition that physical activity is beneficial but very difficult to maintain over time because of several factors, such as lack of motivation:

It has been very difficult for me lately to exercise, getting on the bike is very difficult because I feel that it is like something psychological and I am going to get tired

Survivor #14

Survivors of cancer also reported engaging in less physical activity as they get further away from their initial cancer diagnosis:

I used to exercise at the beginning, but right now I don’t do it, I used to go out for a walk but now I don’t

Survivor #13

Participants also expressed concerns regarding the safety of engaging in physical activity where they live, “It is difficult to find a safe place to walk” (Survivor #14), whereas a participant on the US side of the border had safety issues related to wildlife such as “javelinas [native wild pig-like animal] and snakes” (Survivor #10) in surrounding areas where she usually exercises.

##### Awareness of Cancer Etiology

Survivors mentioned in the context of cancer recurrence awareness of modifiable and nonmodifiable risk factors for cancer (ie, genetics, sex, and diet) and how this contributes to motivation for engaging in healthy lifestyle behaviors. For example, Survivor #6 particularly mentioned, “In my case, the cancer I got was genetic,” whereas Survivor #1 referred to her cancer as being hormone-based, “In my case, my cancer is very hormone-influenced.” Given this awareness, survivors of cancer understood not only the ongoing risk for recurrence but also the repercussions for their family members, “Family is also more likely to get cancer and that gives motivation for everyone to stay healthy” [Survivor #3]. Survivors of cancer reported on knowing the value of following recommendations and guidelines for cancer surveillance and prevention, “In my checkups that I get done every six months, thank God everything has looked good” [Survivor #16].

##### Survivor of Cancer Support Network

Participants reported they would like to meet with other survivors of cancer to share their experiences, from physical effects of the treatment to sharing information on other lifestyle factors such as dietary changes and recipes:

Being in a group where we all have the same illness, we are all going to talk, we are going to hear opinions of others...give information about how it was or what it has felt, there we see that we are not all the same

Survivor #13

Similarly, another noted, “What motivated me a lot were the cancer survivor classes I attended, I liked it a lot,” (Survivor #5) highlighting how other survivors of cancer are indeed a source of support and motivation.

#### Psychosocial and Physical Effects

##### Physical and Emotional Support

Survivors of cancer reported that the primary way they received support from family was through acts of service. Survivors reported that delegating responsibilities to their partners and children was imperative while navigating their cancer treatment:

During my second treatment, I was bed-bound and felt sicker, therefore, I learned I had to delegate responsibilities to my daughters, who have always been there, and my husband

Survivor #10

These included responsibilities within the household (ie, cooking and cleaning):

My children come and cook for us, often we have a barbecue and we spend time with them...my husband takes care of the grocery shopping

Survivor #5

Survivors also expressed that family members played a significant role in providing emotional support and motivation:

My children and grandchildren with me motivation to do what I have to do even when I am not in the mood to do it

Survivor #10

##### Family Support for a Healthy Lifestyle

Participants reported that family provided a significant amount of support in engaging in health-promoting behaviors. Several survivors of cancer expressed that when it came to physical activity, family members would also engage in physical activity with them:

My husband and my son are the ones that go with me on walks, my son runs with me as well

Survivor #2

Oftentimes, the support of family in physical activity was crucial for the survivor of cancer to engage in physical activity:

Once my husband gets home from work, we go for a walk outside for like a mile or a mile and a half

Survivor #2

Support for healthier eating habits was different from that for physical activity. Many survivors of cancer reported feeling less support from family members when it was related to adopting healthier eating habits than physical activity:

When you are around your family or other people, what they eat is what you are going to have to eat

Survivor #1

Oftentimes, survivors reported that family members have different food preferences that may not align with their goal of adopting healthy dietary habits and that it was difficult to cater to everyone in the family:

My husband likes fried foods, he loves all those fried foods, therefore, cooking foods for him or for someone else in the house based on their preferences is a barrier because you can’t cook three different dishes at the same time

Survivor #1

##### Importance of Spirituality or Religion

Spirituality or religion provided survivors both motivation to live a healthy life and as a way to cope with their cancer diagnosis:

I do know that it is very important that my God thinks it was worth to let me live one more year, and to think it is worth letting me live another one so I constantly evaluate myself

Survivor #6

Participants often had statements or interjections nested within other themes with a contextual religious lens through which they were looking at their experiences such as “thank God” (Survivor #2) or “God willing” (Survivor #10).

##### Cancer Treatment-Related Symptoms Interfere With Lifestyle

Lymphedema was the most commonly reported physical effect during the interviews among survivors. Participants often reported the ongoing struggle of dealing with lymphedema in activities of daily living and influencing ability to engage in physical activity. One survivor mentioned:

There are things that, for example, I can no longer lift, things that I used to lift heavy or things like that...now I’m trying to regain that mobility in my life but I know that maybe I won’t get it back 100% due to lymphedema

Survivor #1

The connection between physical activity and minimizing the effects of lymphedema was also mentioned by the participants. There were no other mentions of coexisting chronic conditions.

##### Financial Barriers to Live a Healthy Lifestyle

Survivors of cancer reported that financial difficulties were a major barrier to engaging in a healthy lifestyle:

Having more economic help would help me relax given I am a single person...I have been working for myself and my own wellbeing

Survivor #14

With regard to diet, many participants held the belief that eating healthy is more expensive and identified lack of finances as a barrier for acquiring healthful foods:

We know that leading a healthy lifestyle is always a little more expensive because we have to buy vegetables

Survivor #16

Similarly, another participant reported that oftentimes fast food can be cheaper:

Many times the food that we have to eat or have to prepare is more expensive than buying junk food

Survivor #3

Likewise, financial barriers were reported to engage in physical activity. Participants reported that financial difficulties prevented them from acquiring or accessing equipment or spaces for physical activity, “It has been a while since we are trying to buy a treadmill and I still can’t buy it” (Survivor #2) and “Having money to buy what we have to in order to lead a healthy lifestyle...like going to the gym” (Survivor #16).

### Qualitative Results for Caregivers

#### Caregiving Experience Effect on Lifestyle Habits

Caregivers reported that seeing survivors of cancer navigating cancer treatment increased their awareness and influenced them to engage in healthier lifestyle behaviors themselves. For example, participants reported increasing physical activity levels and making additional efforts to eat healthy with the purpose of “keeping the cancer away” (Caregiver #3) not only for themselves but also within their families: “If we all eat healthy and exercise, the cancer won’t be back in our family” (Caregiver #3) (original quotes in Spanish are available in Multimedia Appendix 2). Smoking cessation was acknowledged as an important aspect in maintaining a healthy lifestyle and one caregiver reported quitting smoking after their loved one was diagnosed:

Me for example, before the cancer treatment, I used to smoke cigarettes, but from the moment of diagnosis, that is when I changed this habit because I realized I was hurting myself.

Caregiver #3

Cancer diagnosis also had a positive effect on other cancer prevention screening measures among caregivers. For one caregiver in their family, being proactive and engaging in precautionary measures for cancer prevention was a new adopted habit:

Before I did not check myself and now I go once a year, I always go and have my ultrasound, my mammogram, or I go with them [sisters who are survivors of cancer] when they have a check-up and yes, a disease like this affecting your family it really changes your life.

Caregiver #10

Although the cancer experience of a loved one was reported to be a source of motivation for improving their own lifestyle, caregivers also reported a decreased effect over time. For example, Caregiver #3 mentioned that although they were caring for the survivor of cancer, they were more diligent in engaging in healthy lifestyle habits, whereas now, the fact that survivors of cancer and caregivers have their own separate routines has diminished this effect.

#### Supporting the Survivor

Providing support for survivors, particularly by taking care of activities related to their daily living, was commonly reported. Daily routines changed during their loved ones’ cancer treatment to provide support for activities of daily living and emotional needs to manage side effects (ie, hair loss, lack of appetite, or mood). Oftentimes, there was a balance between responsibilities and time:

When I was taking care of my mother, I would get up much earlier than normal to be able to take care of my responsibilities; go to drop off the children at school and come back so that I could help [my mom] clean her house, make her breakfast, and when she had the surgery, I would change her dressings

Caregiver #3

One caregiver reported it is crucial to:

give them a lot of affection so that they do not feel alone, that all things happen and that this will definitely happen but above all give them a lot of love, a lot of trust, a lot of assurance that they are not alone

Caregiver #4

Importantly, caregivers highlighted their experience and caregiving duties to be an honor rather than a burden, one considered it, “gratifying for oneself” and highlighted that:

We do not know if tomorrow one may be in the same situation and I want to think that there will always be someone you can count on

Caregiver #8

## Discussion

### Principal Findings

This is the first qualitative study to explore Mexican-origin survivors of breast cancer and caregivers’ perspectives on lifestyle behaviors, namely, diet and physical activity, and important aspects for developing and adapting a culturally relevant lifestyle intervention for survivors of cancer residing in border communities. Using a community-based participatory approach between an academic partner and a community partner enabled us to recruit and conduct interviews with 12 survivors of breast cancer and 7 caregivers. The perspectives of bilingual and bicultural members of our team allowed us to identify relevant and culturally specific perspectives related to the impact of cancer on lifestyle, perceptions of lifestyle, and intervention content and delivery preferences. Key themes, which emerged through framework guided directed thematic analysis, included health promotion and psychosocial and physical effects among survivors of cancer, whereas only health promotion was a key theme among caregivers. Family was a central and recurring subtheme throughout as was the desire for access to content experts for health promotion education.

Participants expressed an awareness of the role of nutrition and physical activity in cancer prevention and survivorship. Physical activity was linked with physical effects (where physical activity improved side effects related to cancer treatment and side effects interrupted abilities to engage in physical activity) and was considered more challenging to engage in compared with other recommended lifestyle habits (eg, diet). Family provided physical and emotional support, and healthy lifestyle behaviors (or lack thereof) were experienced as a family unit. Variations were noted among caregivers, including that the cancer diagnosis was influential to their lifestyle behaviors and that there was pride in supporting the survivor. Unique to this study was the self-reported diet and physical activity before and after treatment in both the survivor and caregivers, which provided quantitative support for our qualitative findings related to lifestyle behaviors.

Our findings revealed an emphasis on what not to eat and a reported lack of reliable information. Diet plays an important role in Mexican culture, with traditions and social components centered on planning, cooking, and eating foods [34]. Participants in this study reported eating typical Mexican foods with family and friends as a common quality time activity with loved ones. However, Mexican food was commonly perceived as unhealthy and was labeled as a barrier for healthy eating because of its *very greasy* nature. Concordant findings were reported by Ramirez et al [35], where participants reported that “growing up in a Mexican household, a lot of the foods that [they] eat aren’t very healthy.” Additional findings from the study by Ramirez et al [35] described how typical Mexican foods (ie, pozole and tamales) are perceived as foods eaten as a *treat* and are not foods that constitute a healthy eating pattern. Interestingly, several reports in the literature have focused on describing and investigating a *traditional Mexican diet* and its effects on health [36-40], which contrast with the reported belief of the overall Mexican diet being unhealthy, as observed in our study and others. Overall, a traditional Mexican diet is characterized by higher amounts of plant-based foods, including, but not limited to, legumes, grains, and vegetables and high amount of specific foods such as maíz (corn), beans, chile, squash, onion, and garlic [41]. A traditional Mexican dietary pattern has been associated with lower systemic inflammation, lower insulin sensitivity, lower risk for overweight and obesity, and lower risk of obesity-related cancer mortality collectively across several studies, including a randomized controlled feeding trial and other epidemiological studies [36-40]. Importantly, the participants in our study reported several strategies that would help them overcome reported barriers to healthy eating. Among the top requests, having access to an informed nutrition professional and participating in survivorship support groups were highly requested. Such strategies should be considered as future dietary intervention components among border-dwelling Mexican-origin survivors of cancer.

The Quality of Cancer Survivorship Care Framework [18] used to analyze our interviews can be applied to intervention design as well to include tailored risk assessment for behaviors, symptoms, finances, and interpersonal relationships in addition to education provided by clinical professionals and care coordination. Our results further emphasized the need for reliable information related to diet and physical activity and the importance of content knowledge experts, such as registered dietitians, to be involved in intervention planning and delivery. Engagement of culturally competent knowledge experts throughout intervention design and delivery is a way to provide reliable education and increase participants’ acceptability and access to clinical professionals. As the experience of physical effects and comorbidities is heterogeneous in survivorship, access to clinical professionals may be required to provide tailored advice on how to safely engage in physical activity and dietary change. Important considerations for intervention planning include the perceived costs observed in this study related to engaging and maintaining a healthy lifestyle and feelings of safety around physical activity.

Highlighted by the intersection of the survivor, their family connections, and desire for community and structural support for a healthy lifestyle, delivery of an intervention could further benefit from theoretical guidance from the socioecological model. Theoretical constructs are associated with meeting current diet and physical activity recommendations among survivors of cancer, and theory-informed interventions often have a sustained impact on behavior change in survivors of cancer [42,43]. The socioecological model describes the complex interactions between individuals nested within relationships and environments. Successful interventions built from the socioecological model include components of education, skills enhancement, modifications to home or institutional environments, community capacity, and policy advocacy [44] and inclusion of the survivorship community (including survivors of cancer, caregivers, providers, advocates, public health professionals, and policy makers) [45]. The socioecological model may promote better outcomes for survivors of cancer [45].

### Strengths and Limitations

Strengths of this study include the value of a participatory approach. The effective collaboration between the investigating team and community partners allowed for innovative and successful completion of the study, particularly in a highly unpredictable time such as early during the COVID-19 pandemic. This approach allowed the development of strategies that were feasible for implementation among study participants, such as conducting and completing all study-related activities remotely and troubleshooting technologic difficulties that resulted in little to no issues with participants accessing the videoconferencing software and further expanded our reach to rural survivors of cancer. This study was limited by the breast cancer perspective only and may not be translated to all obesity-related cancers or the cancer experience in male survivors of cancer. In addition, caregiver experiences may be limited in their generalizability to other cancer populations. The Quality of Cancer Survivorship Care Framework [18] used to guide our analysis did not translate seamlessly into caregiver interviews, and some of the themes from the framework were minimal in our results. This warrants for the use of a caregiver specific framework, such as the one developed by Fletcher et al [46], which seeks to incorporate relevant factors for this population such as culture, socioeconomic status, and access to health care when analyzing data as the one presented in this qualitative study.

### Recommendations for Intervention Planning and Next Steps

In summary, the future development of culturally appropriate and acceptable lifestyle interventions to improve cancer survivorship among Hispanic populations living in the United States–Mexico border may consider the following points:

1. Culturally relevant and competent content consisting of traditional Mexican diet and social norms
2. Assess and address barriers, including physical and psychological side effects of cancer treatment and environmental and financial factors
3. Multimodal programming composed of written educational materials, interactive support groups, and nutrition or physical activity content experts
4. Inclusion of a survivor-identified informal caregiver or family members
5. Implementation strategies to investigate integration into a clinical setting with delivery by CHWs

Acknowledging the heterogeneity of the Hispanic population, studies should focus on programs that target populations from different countries of origin and regions of the United States separately to address the specific lifestyle behavior needs and barriers to the target population and evaluate which cultural adaptations are well received and effective in improving feasibility, acceptability, and replication of culturally tailored programs. Given that different cancer sites and treatments may yield distinct complications and side effects, cancer diagnosis, treatment, and subsequent treatment-related side effects influence on lifestyle behaviors should be considered. Forming or continuing community partnerships and collaboration can establish effective and sustainable lifestyle behavior programming for advancing cancer survivorship among Hispanic populations.

### Conclusions

Our study identified that Mexican-origin survivors of breast cancer desire relevant and evidence-based information related to healthy lifestyle behaviors and highlight the influence of family and community on the adoption of a healthy diet and physical activity habits. These qualitative findings and recommendations support a theory-informed, evidenced-based, CHW-led, culturally relevant lifestyle program to reduce the burden of cancer recurrence, comorbidities, and potential outcomes after cancer in Mexican-origin survivors of cancer living in border communities and cancer prevention among cancer caregivers.

## Appendix

Multimedia Appendix 1 Identified themes and quotes among survivors of cancer (n=12).

Multimedia Appendix 2 Identified themes and quotes among caregivers (n=7).

## Abbreviations

CHW: community health worker
MCHC: Mariposa Community Health Center

## References

[1] (2018). American Cancer Society Cancer Facts & Figures for Hispanics/Latinos 2018-2020.

[2] Felicetti F, Fortunati N, Brignardello E (2018). Cancer survivors: an expanding population with an increased cardiometabolic risk. Diabetes Res Clin Pract.

[3] Roy S, Vallepu S, Barrios C, Hunter K (2018). Comparison of comorbid conditions between cancer survivors and age-matched patients without cancer. J Clin Med Res.

[4] Schoormans D, Vissers PA, van Herk-Sukel MP, Denollet J, Pedersen SS, Dalton SO, Rottmann N, van de Poll-Franse L (2018). Incidence of cardiovascular disease up to 13 year after cancer diagnosis: a matched cohort study among 32 757 cancer survivors. Cancer Med.

[5] Dominguez K, Penman-Aguilar A, Chang MH, Moonesinghe R, Castellanos T, Rodriguez-Lainz A, Schieber R, Centers for Disease Control and Prevention (CDC) (2015). Vital signs: leading causes of death, prevalence of diseases and risk factors, and use of health services among Hispanics in the United States - 2009-2013. MMWR Morb Mortal Wkly Rep.

[6] Hales CM, Carroll MD, Fryar CD, Ogden CL (2017). Prevalence of obesity among adults and youth: United States, 2015-2016. NCHS Data Brief.

[7] Centers for Disease Control and Prevention (CDC) (2004). Prevalence of diabetes among Hispanics--selected areas, 1998-2002. MMWR Morb Mortal Wkly Rep.

[8] Campos CL, Rodriguez CJ (2019). High blood pressure in Hispanics in the United States: a review. Curr Opin Cardiol.

[9] Paulozzi LJ, McDonald JA, Sroka CJ (2021). A disparity beneath a paradox: cancer mortality among young Hispanic Americans in the US-Mexico border region. J Racial Ethn Health Disparities.

[10] (2017). The U.S.-Mexico border region. U.S. Department of Health & Human Services.

[11] Salinas JJ, Su D, Al Snih S (2013). Border health in the shadow of the Hispanic paradox: issues in the conceptualization of health disparities in older Mexican Americans living in the Southwest. J Cross Cult Gerontol.

[12] Norman SA, Potashnik SL, Galantino ML, De Michele AM, House L, Localio AR (2007). Modifiable risk factors for breast cancer recurrence: what can we tell survivors?. J Womens Health (Larchmt).

[13] Thomson CA, Rock CL, Thompson PA, Caan BJ, Cussler E, Flatt SW, Pierce JP (2011). Vegetable intake is associated with reduced breast cancer recurrence in tamoxifen users: a secondary analysis from the Women's Healthy Eating and Living Study. Breast Cancer Res Treat.

[14] Lim JW, Gonzalez P, Wang-Letzkus MF, Ashing-Giwa KT (2009). Understanding the cultural health belief model influencing health behaviors and health-related quality of life between Latina and Asian-American breast cancer survivors. Support Care Cancer.

[15] Montoya JA, Salinas JJ, Barroso CS, Mitchell-Bennett L, Reininger B (2011). Nativity and nutritional behaviors in the Mexican origin population living in the US-Mexico border region. J Immigr Minor Health.

[16] White A, Pollack LA, Smith JL, Thompson T, Underwood JM, Fairley T (2013). Racial and ethnic differences in health status and health behavior among breast cancer survivors--behavioral risk factor surveillance system, 2009. J Cancer Surviv.

[17] Nayak P, Paxton RJ, Holmes H, Thanh Nguyen H, Elting LS (2015). Racial and ethnic differences in health behaviors among cancer survivors. Am J Prev Med.

[18] Nekhlyudov L, Mollica MA, Jacobsen PB, Mayer DK, Shulman LN, Geiger AM (2019). Developing a quality of cancer survivorship care framework: implications for clinical care, research, and policy. J Natl Cancer Inst.

[19] Oster NV, Welch V, Schild L, Gazmararian JA, Rask K, Spettell C (2006). Differences in self-management behaviors and use of preventive services among diabetes management enrollees by race and ethnicity. Dis Manag.

[20] Orzech KM, Vivian J, Huebner Torres C, Armin J, Shaw SJ (2013). Diet and exercise adherence and practices among medically underserved patients with chronic disease: variation across four ethnic groups. Health Educ Behav.

[21] Onwudiwe NC, Mullins CD, Winston RA, Shaya FT, Pradel FG, Laird A, Saunders E (2011). Barriers to self-management of diabetes: a qualitative study among low-income minority diabetics. Ethn Dis.

[22] Zeh P, Sandhu HK, Cannaby AM, Sturt JA (2014). Cultural barriers impeding ethnic minority groups from accessing effective diabetes care services: a systematic review of observational studies. Divers Equal Health Care.

[23] Narayan MC (2002). The national standards for culturally and linguistically appropriate services in health care. Care Manag J.

[24] Estrada RD, Messias DK (2015). A scoping review of the literature: content, focus, conceptualization and application of the national standards for culturally and linguistically appropriate services in health care. J Health Care Poor Underserved.

[25] Colorafi KJ, Evans B (2016). Qualitative descriptive methods in health science research. HERD.

[26] Teufel-Shone NI, Schwartz AL, Hardy LJ, de Heer HD, Williamson HJ, Dunn DJ, Polingyumptewa K, Chief C (2018). Supporting new community-based participatory research partnerships. Int J Environ Res Public Health.

[27] (2019). QuickFacts: Nogales City, Arizona. United States Census Bureau.

[28] Pérez LM, Martinez J (2008). Community health workers: social justice and policy advocates for community health and well-being. Am J Public Health.

[29] Thomson CA, Overholser LS, Hébert JR, Risendal BC, Morrato EH, Wheeler SB (2021). Addressing cancer survivorship care under COVID-19: perspectives from the cancer prevention and control research network. Am J Prev Med.

[30] Newman VA, Thomson CA, Rock CL, Flatt SW, Kealey S, Bardwell WA, Caan BJ, Pierce JP, Women's Healthy Eating and Living (WHEL) Study Group (2005). Achieving substantial changes in eating behavior among women previously treated for breast cancer--an overview of the intervention. J Am Diet Assoc.

[31] Centers for Disease Control and Prevention (U.S.), Office for the Associate Director of Communication, Division of Communication Services (2012). Cultural insights: communicating with Hispanics/Latinos. Centers for Disease Control and Prevention.

[32] (2021). Behavioral measurement and interventions shared resource. University of Arizona.

[33] Hsieh HF, Shannon SE (2005). Three approaches to qualitative content analysis. Qual Health Res.

[34] Lindberg NM, Stevens VJ, Halperin RO (2013). Weight-loss interventions for Hispanic populations: the role of culture. J Obes.

[35] Ramírez AS, Golash-Boza T, Unger JB, Baezconde-Garbanati L (2018). Questioning the dietary acculturation paradox: a mixed-methods study of the relationship between food and ethnic identity in a group of Mexican-American women. J Acad Nutr Diet.

[36] Flores M, Macias N, Rivera M, Lozada A, Barquera S, Rivera-Dommarco J, Tucker KL (2010). Dietary patterns in Mexican adults are associated with risk of being overweight or obese. J Nutr.

[37] Santiago-Torres M, Tinker LF, Allison MA, Breymeyer KL, Garcia L, Kroenke CH, Lampe JW, Shikany JM, Van Horn L, Neuhouser ML (2015). Development and use of a traditional Mexican diet score in relation to systemic inflammation and insulin resistance among women of Mexican descent. J Nutr.

[38] Santiago-Torres M, Kratz M, Lampe JW, Tapsoba JD, Breymeyer KL, Levy L, Villaseñor A, Wang CY, Song X, Neuhouser ML (2016). Metabolic responses to a traditional Mexican diet compared with a commonly consumed US diet in women of Mexican descent: a randomized crossover feeding trial. Am J Clin Nutr.

[39] Murtaugh MA, Sweeney C, Giuliano AR, Herrick JS, Hines L, Byers T, Baumgartner KB, Slattery ML (2008). Diet patterns and breast cancer risk in Hispanic and non-Hispanic white women: the Four-Corners Breast Cancer Study. Am J Clin Nutr.

[40] Lopez-Pentecost M, Crane TE, Garcia DO, Kohler LN, Wertheim BC, Hebert JR, Steck SE, Shivappa N, Santiago-Torres M, Neuhouser ML, Hatsu IE, Snetselaar L, Datta M, Kroenke CH, Sarto GE, Thomson CA (2020). Role of dietary patterns and acculturation in cancer risk and mortality among postmenopausal Hispanic women: results from the Women’s Health Initiative (WHI). J Public Health.

[41] Valerino-Perea S, Lara-Castor L, Armstrong ME, Papadaki A (2019). Definition of the traditional Mexican diet and its role in health: a systematic review. Nutrients.

[42] Skiba MB, Jacobs ET, Crane TE, Kopp LM, Thomson CA (2021). Relationship Between Individual Health Beliefs and Fruit and Vegetable Intake and Physical Activity Among Cancer Survivors: Results from the Health Information National Trends Survey. J Adolesc Young Adult Oncol.

[43] Stacey FG, James EL, Chapman K, Courneya KS, Lubans DR (2015). A systematic review and meta-analysis of social cognitive theory-based physical activity and/or nutrition behavior change interventions for cancer survivors. J Cancer Surviv.

[44] Golden SD, Earp JA (2012). Health Educ Behav.

[45] Moore AR, Buchanan ND, Fairley TL, Lee Smith J (2015). Public health action model for cancer survivorship. Am J Prev Med.

[46] Fletcher BS, Miaskowski C, Given B, Schumacher K (2012). The cancer family caregiving experience: an updated and expanded conceptual model. Eur J Oncol Nurs.

