# Supplementary material for: Health Promotion Among Mexican-Origin Survivors of Breast Cancer and Caregivers Living in the United States–Mexico Border Region: Qualitative Analysis From the Vida Plena Study
Source: JMIR Cancer. 2022 Feb 24;8(1):e33083. doi: 10.2196/33083 (PMC8914737; doi:10.2196/33083)
Supplement: Multimedia Appendix 1 [file cancer_v8i1e33083_app1.docx]

**Multimedia Appendix 1.** Identified themes and quotes among survivors of cancer (n=12).

| Domain and theme | | | Subtheme | Quote (Spanish) | Quote (English) |
| --- | --- | --- | --- | --- | --- |
| **Health promotion** | | | | | |
|  | **Diet and nutrition** | | | | |
|  |  | The unhealthy Mexican diet | | - “Yo creo que la barrera más grande en cuanto a la cultura de donde yo vengo es la alimentación que se nos ha inculcado, el estilo de los alimentos son muy grasosos a veces en todos los platillos mexicanos.” [Sobreviviente #1] | - “I believe that the biggest barrier in terms of the culture where I come from is the food that has been instilled in us, the style of food is very, very fatty sometimes, all Mexican dishes are very fatty.” [Survivor #1] |
|  |  | Eating on time important for healthy eating | | - **“**Si quieres llevar una vida saludable deberías comer desayuno exactamente a las 8 de la mañana, comer a las 2 de la tarde, y cenar a las 8 de la noche.” [Sobreviviente #14] | - “If you want to live a healthy life you should eat breakfast at exactly 8 in the morning, eat at 2 in the afternoon, and have dinner at 8 at night.” [Survivor #14] |
|  |  | Reducing or avoiding certain foods to eat healthy | | - “Yo digo nomás que uno debe de comer menos de todo, por ejemplo, yo ya tengo muchos años que nomás me como dos tortillas y ya no cómo más.” [Sobreviviente #2] | - **“**I think one should eat less of everything, for example for many years I have been eating just two tortillas and that is it.” [Survivor #2] |
|  |  | Needing reliable nutrition and diet-related information for themselves and their family members | | - “Siempre he pensado que a las sobrevivientes y a las personas que entran en tratamiento de cáncer, así como nos mandan a la terapia física después de nuestras cirugías y todo eso, también debería de existir una clase o que nos manden con el nutriólogo.” [Sobreviviente #1] - “Hay que reeducador a toda la familia...no he encontrado algo así de apoyo a la familia para cambiar el hábito alimenticio, entonces me toca a mí ir a educarlos.” [Sobreviviente #1] | - “I have always thought that people who enter cancer treatment and survivors, just like they send us to physical therapy after our surgeries, they should also send us to a nutrition class or to the nutritionist.” [Survivor #1] - “We have to re-educate the whole family...I have yet to find resources that provide support to the family to change the eating habits, therefore it is up to me to educate them.” [Survivor #1] |
|  | **Physical activity** | | | | |
|  |  | Engaging in physical activity to manage physical effects after cancer treatment | | - “Ahora se me hace más necesario el ejercicio porque el brazo, pues ya ves que me quedo entumido, y si no estoy haciendo ejercicio se me pone pesado y yo lo siento hinchado...entonces tengo que hacer ejercicio para quitar esa sensación.” [Sobreviviente #11] | - “Exercise is now more necessary for me because my arm, well, you can see that I get numb, and if I am not exercising it becomes heavy and I feel swollen...then I have to exercise to remove that feeling.” [Survivor #11] |
|  |  | Decreasing physical activity gradually over time | | - “El ejercicio pues realmente sí lo hice muy al principio, pero ya ahorita no lo hago, antes salía a caminar, pero pues ya ahorita no.” [Sobreviviente #13] | - “I used to exercise at the beginning, but right now I don’t do it, I used to go out for a walk but now I don’t.” [Survivor #13] |
|  | **Cancer prevention** | | | | |
|  |  | Awareness of cancer etiologies and | | - “En mi caso el cáncer que yo he tenido ha sido genético.” [Sobreviviente #6] - “En mi caso el cáncer puede ser muy hormonal.” [Sobreviviente #1] | - “In my case, the cancer I got was genetic” [Survivor #6] - “In my case, my cancer is very hormone-influenced.” [Survivor #1] |
|  |  | High cancer risk for family members | | - “La familia [también] es más propensa a tener un cáncer y yo creo que la motivación de eso es el simple hecho de mantener a todos saludables.” [Sobreviviente #3] | - “The family is also more likely to get cancer and that gives motivation for everyone to stay healthy.” [Survivor #3] |
|  |  | Importance of cancer surveillance strategies in cancer recurrence prevention | | - “En mis estudios que me hacen cada seis meses gracias a Dios he salido muy bien de todo.” [Sobreviviente #16] | - “In my checkups that I get done every six months, thank God everything has looked good.” [Survivor #16] |
|  |  | Importance of cancer survivorship groups | | - “Estar en un grupo donde todos tenemos el mismo mal, todos vamos a platicar, vamos a oír opiniones de los demás,...dar información de cómo le fue o qué ha sentido, ahí vemos que no todos somos iguales.” [Sobreviviente #13] - **“**Lo que me motivaba mucho eran las clases que iba del grupo de cáncer, a mí me gustaba mucho.” [Sobreviviente #5] | - “Being in a group where we all have the same illness, we are all going to talk, we are going to hear opinions of others,...give information about how it was or what it has felt, there we see that we are not all the same.” [Survivor #13] - “What motivated me a lot were the cancer survivor classes I attended, I liked it a lot.” [Survivor #5] |
| **Psychosocial effects** | | | | | |
|  | **Family** | | | | |
|  |  | Acts of service from family members as an important source of support | | - “En mi segundo tratamiento sí estuve cama y me sentí un poco mal entonces aprendí a delegar obligaciones o deberes a mis hijas, que siempre han estado pendiente, y mi esposo.” [Sobreviviente #10] | - “During my second treatment, I was bed-bound and felt sicker, therefore, I learned I had to delegate responsibilities to my daughters, who have always been there, and my husband.” [Survivor #10] |
|  |  | A significant role played by family members in providing emotional support | | - “La motivación que me dan mis hijos y mis nietos también para hacer las cosas que debo de hacer, aunque a veces no tenga ganas de hacerlo.” [Sobreviviente #10] | - “My children and grandchildren with me motivation to do what I have to do even when I am not in the mood to do it.” [Survivor #10] |
|  |  | Support from family to engage in physical activity | | - “Sí pues mi esposo es el que hace ejercicio conmigo y mi hijo, ellos son los que salen conmigo a caminar mi hijo a correr aquí mismo conmigo.” [Sobreviviente #2] | - “My husband and my son are the ones that go with me on walks, my son runs with me as well.” [Survivor #2] |
|  |  | Less support provided by family to engage in healthy eating habits | | - “Cuando tú estás con tu familia o gente que te rodea es lo que vas a tener que comer.” [Sobreviviente #1] - “A mi esposo le encantan las comidas fritas, le fascina todo ese tipo de alimentación, entonces el hacer alimentos para él o para otro en la casa que le guste es también un obstáculo pues no te puedes poner a ser como tres platillos al mismo tiempo.” [Sobreviviente #1] | - “When you are around your family or other people, what they eat is what you are going to have to eat.” [Survivor #1] - “My husband likes fried foods, he loves all those fried foods, therefore, cooking foods for him or for someone else in the house based on their preferences is a barrier because you can’t cook three different dishes at the same time.” [Survivor #1] |
|  | **Spirituality/Religion** | | | | |
|  |  | Spirituality/religion as a source of motivation after cancer diagnosis | | - “Yo sí sé que es muy importante que mi Dios dijera valió la pena dejarte [vivir] un año más y te voy a dejar el otro a ver cómo lo haces entonces me evalúo constantemente.” [Sobreviviente #16] | - “I do know that it is very important that my God thinks it was worth to let me live one more year, and to think it is worth letting me live another one so I constantly evaluate myself.” [Survivor #16] |
|  | **Financial impact** | | | | |
|  |  | Financial barriers to acquiring healthy foods | | - “Sabemos que llevar un estilo de vida saludable siempre es un poquito más caro porque tenemos que comprar verduras.” [Sobreviviente #16] - **“**Muchas veces los alimentos que tenemos que consumir o tenemos que preparar son más costosos que comprar una comida chatarra.” [Sobreviviente #3] | - “We know that leading a healthy lifestyle is always a little more expensive because we have to buy vegetables.” [Survivor #16] - “Many times the food that we have to eat or have to prepare is more expensive than buying junk food.” [Survivor #3] |
|  |  | Financial barriers to accessing equipment or spaces for physical activity | | - “Desde cuando según estamos comprando una caminadora y no todavía no la puedo comprar.” [Sobreviviente #2] - **“**El tener nosotros dinero para poder comprar lo que tenemos que para llevar un estilo de vida saludable...y todo eso como asistir al gym.” [Sobreviviente #16] | - “It has been a while since we are trying to buy a treadmill and I still can’t buy it.” [Survivor #2] - “Having money to buy what we have to in order to lead a healthy lifestyle...like going to the gym.” [Survivor #16] |
| **Physical effects** | | | | | |
|  | **Cancer treatment side effects** | | | | |
|  |  | Interference of symptoms after cancer treatment with lifestyle | | - “Hay cosas que por ejemplo ya no puedo levantar, cosas que levantaba antes pesadas o cosas así...ahora recuperar esa movilidad en mi vida y saber que a la mejor no la obtengo al 100 por el linfedema.” [Sobreviviente #1] | - “There are things that, for example, I can no longer lift, things that I used to lift heavy or things like that...now I’m trying to regain that mobility in my life but I know that maybe I won’t get it back 100% due to lymphedema.” [Survivor #1] |
