# Supplementary material for: Health Promotion Among Mexican-Origin Survivors of Breast Cancer and Caregivers Living in the United States–Mexico Border Region: Qualitative Analysis From the Vida Plena Study
Source: JMIR Cancer. 2022 Feb 24;8(1):e33083. doi: 10.2196/33083 (PMC8914737; doi:10.2196/33083)
Supplement: Multimedia Appendix 2 [file cancer_v8i1e33083_app2.docx]

**Multimedia Appendix 2.** Identified themes and quotes among caregivers (n=7).

| Domain and theme | | Subtheme | Quote (Spanish) | Quote (English) |  |
| --- | --- | --- | --- | --- | --- |
| **Health promotion** | | | | |  |
|  | **Diet and physical activity lifestyle habits** | | | |  |
|  |  | Cancer survivor’s experience increased caregiver awareness and motivation for healthy living | - Si todos comemos sano y si todos hacemos ejercicio el cáncer en mi familia no va a regresar.” [Cuidador #3] | - “If we all eat healthy and exercise, the cancer won’t be back in our family.” [Caregiver #3] |  |
|  | **Cancer prevention** | | | |  |
|  |  | Motivation to reduce cancer-related behaviors after the survivor’s diagnosis | - “Yo por ejemplo antes del tratamiento fumaba cigarros y del diagnóstico para acá, fue cuando a mí en lo personal cambie mucho en este sentido porque realmente me estaba haciendo daño a mí mismo.” [Cuidador #3] | - “Me for example, before the cancer treatment, I used to smoke cigarettes, but from the moment of diagnosis, that is when I changed this habit because I realized I was hurting myself.” [Caregiver #3] |  |
|  |  | Motivation to engaging in cancer prevention behaviors | - “Yo antes no me revisaba y ahora ya voy una vez al año, siempre voy y me hago mi ultrasonido mi mamografía, o las acompañó a ellas [las sobrevivientes de cáncer] cuando tienen algo chequeo que hacer y si, realmente te cambia la vida que una enfermedad como ésta pase por tu familia.” [Cuidador #10] | - “Before I did not check myself and now I go once a year, I always go and have my ultrasound, my mammogram, or I go with them [sisters who are cancer survivors] when they have a check-up and yes, a disease like this affecting your family it really changes your life.” [Caregiver #10] |  |
| **Psychosocial effects** | | | | |  |
|  | **Family** | | | |  |
|  |  | Providing support to the survivor through acts of service | - “Cuando estaba cuidando a mi mamá me levantaba mucho más temprano de lo normal para poder hacer las cosas que me correspondía; ir a dejar a los niños a la escuela y regresarme para poderla ayudar a limpiar su casa hacerle desayuno, en el momento que está operada era pues hacerle su curación, y hacerles el desayuno específico.” [Cuidador #3] | - “When I was taking care of my mother, I would get up much earlier than normal to be able to take care of my responsibilities; go to drop off the children at school and come back so that I could help [my mom] clean her house, make her breakfast, and when she had the surgery, I would change her dressings.” [Caregiver #3] |  |
|  |  | Importance of providing emotional support to the survivor | - “[es importante] Darles mucho cariño que no se sientan solas, que todas las cosas pasan y que lo eso va a pasar definitivamente, pero sobre todo darle mucho amor, mucha confianza, mucha seguridad de que no estén solas.” [Cuidador #4] | - “Give them a lot of affection so that they do not feel alone, that all things happen and that this will definitely happen but above all give them a lot of love, a lot of trust, a lot of assurance that they are not alone.” [Caregiver #4] | |
|  |  | Supporting the survivor an honorary action for the caregiver | - “No sabemos si el día de mañana uno caiga en esta situación y yo quiero pensar que va a haber alguien en que puedas contar.” [Cuidador #8] - “[ayudar al sobreviviente] es satisfactorio para uno mismo.” [Cuidador #8] | - “We do not know if tomorrow one may be in the same situation and I want to think that there will always be someone you can count on.” [Caregiver #8] - “[helping the survivor] is satisfying for oneself.” [Caregiver #8] |  |
